# Time trends in pediatric fractures in a Swedish city from 1950 to 2016

**DOI:** 10.1080/17453674.2020.1783484

**Published:** 2020-06-26

**Authors:** Erika Bergman, Vasileios Lempesis, Jan-Åke Nilsson, Lars Jephsson, Björn E Rosengren, Magnus K Karlsson

**Affiliations:** Clinical and Molecular Osteoporosis Research Unit, Department of Clinical Sciences and Orthopedics, Lund University, Skåne University Hospital, Malmö, Sweden

## Abstract

Background and purpose — As previous studies indicate time trends in pediatric fracture incidence, we followed the incidence in a Swedish city between 1950 and 2016.

Patients and methods — Malmö city, Sweden had 322,574 inhabitants in 2015. We used diagnosis registry, charts, and radiographs of the only city hospital to classify fractures in individuals < 16 years in 2014–2016, and compared these with data from 1950–2006. We used joinpoint regression to analyze time trends and present results as mean annual percentage changes (APC). Differences between periods are described as incident rate ratios (IRR). To describe uncertainty, 95% confidence intervals (CI) are used.

Results — During 2014–2016 the pediatric fracture incidence was 1,786 per 10^5^ person-years (boys 2,135 and girls 1,423). From 1950 onwards age-adjusted fracture incidence increased until 1979 in both boys (APC +1.5%, CI 1.2–1.8) and girls (APC +1.6%, CI 0.8–2.5). The incidence remained stable from 1979 to 2016 (APC in boys 0.0%, CI –0.3 to 0.3 and in girls –0.2%, CI –1.1 to 0.7). Age-adjusted incidence 2014–2016 was higher than 2005–2006 in girls (IRR 1.1, CI 1.03–1.3), but not in boys (IRR 1.0, CI 0.9–1.1).

Interpretation — Fracture incidence was in girls higher in 2014–2016 than in 2005–2006. However, only with more than 2 measuring points are meaningful trend analyses possible. When we analyzed the period 1950–2016 with 17 measuring points and joinpoint regression, we found that fracture incidence increased in both sexes until 1979 but has thereafter been stable.

As many as 1/3 of all individuals have sustained a fracture before their 17th birthday (Cooper et al. 2004). However, as there are evident time trends in pediatric fracture incidence (Hedström et al. 2010, Mayranpaa et al. 2010, Jenkins et al. 2018, Koga et al. 2018), an update on occurrence is of interest. Published reports are contradictory. Some reports indicate an increase in pediatric fracture incidence, with higher incidences reported in 2007 than in 1998 in Sweden (Hedström et al. 2010), higher in 1999–2007 than in 1979–1987 in Japan (Koga et al. 2018) and higher in 2015 than in 2005 in Australia (Jenkins et al. 2018). Other authors report a lower incidence in 2005 than in 1983 (Mayranpaa et al. 2010). The discrepancies may be real but may also depend on varying ascertainment methods, time trend differences between regions, or that the years chosen for comparisons were not similar.

In the city of Malmö, Sweden, we have previously found that the unadjusted pediatric fracture incidence was higher in 1979 than in 1950 (Landin 1983), lower in 1993–1994 than in 1975–1979 (Tiderius et al. 1999) and similar in 2005–2006 and 1993–1994 (Lempesis et al. 2017). In contrast, no difference was apparent in the age-adjusted incidence when comparing 1993–1994 with 1976–1979 (Lempesis et al. 2017), indicating that changes in demography between these periods have influenced fracture incidences.

Therefore, we analyzed long-term time trends in pediatric fracture incidence. Secondarily, we compared the present fracture occurrence and fracture etiology with the last previous update a decade ago. Thus, we collected information on fracture occurrence during 2014–2016 in a similar fashion to previous data collection for the period 1950–2006.

## Patients and methods

### Background information

Malmö is a city in southern Sweden which in 2014 had a population of 318,107 inhabitants (58,585 < 16 years of age), 2015: 322,574 (60,519 < 16 years of age) and 2016: 328,494 (62,513 < 16 years of age) (Statistics Sweden 2017). The Skåne University Hospital in Malmö is the only hospital that provides trauma care for the population. There are few private clinics in the city, although some have orthopedic surgeons on their staff. However, these clinics evaluate only scheduled patients, and do not have any emergency service capacity. Fractures in the city are thus diagnosed and treated at the Skåne University Hospital. If a patient with a fracture initially attends a primary care center, the family doctor refers the patient to the Department of Radiology at Skåne University Hospital for evaluation. If the radiographic exam reveals a fracture, the patient is directly transferred to the Department of Emergency at the hospital, a visit that renders a fracture classification.

### Ascertainment of fracture data

All radiographs taken within the general health care system in the region of Skåne, Sweden are, since 2001, when the hospital changed from physical to digital radiographs, filed according to the unique 10-digit national personal identity number of the patient. All radiographs and reports are saved in a digital archive. This digital archive has been used to identify pediatric fractures 2005–2006 (Lempesis et al. 2017). Prior to 2001, all radiographs were organized according to diagnosis, year of injury, and anatomical region in an archive that could be used to identify fractures (Landin 1983, Tiderius et al. 1999). This archive has been used to create a hospital pediatric fracture database that include fractures from 1950, 1955, 1960, 1965, 1970, 1975–1979 (Landin 1983), and 1993–1994 (Tiderius et al. 1999).

In this study we used the same fracture ascertainment method as in 2005–2006 (Lempesis et al. 2017). We searched for pediatric fractures in the digital in- and outpatient diagnosis records at the Departments of Emergency, Orthopedics, Otorhinolaryngology, and Hand Surgery at the hospital. We included records with subsequent criteria: (I) ICD-10 fracture diagnoses S02.3–S02.4, S02.6–S02.9, S12.0–S12.2, S12.7, S22.0–S22.1, S32.0–S32.8, S42.0–S42.9, S52.0–S52.9, S62.0–S62.8, S72.0–S72.9, S82.0–S82.9, and S92.0–S92.9, (II) patient age < 16 years at the time of the fracture event, and (III) city residency in Malmö at the time of the fracture event. During the years 2014–2016, we identified 7,326 visits that fulfilled all 3 criteria. All visits for each patient were then examined in further detail in medical charts, radiographic reports, and referrals to validate fractures. If the fracture diagnosis was ambiguous, radiographs were re-reviewed by the orthopedic surgeon who conducted the review of cases in 2005–2006 (Lempesis et al. 2017), before any verified fracture was included in the study.

For verified fractures, we used the same registration protocol as in our 3 previous reports from Malmö (Landin 1983, Tiderius et al. 1999, Lempesis et al. 2017). We categorized bilateral fractures as 2 separate fractures, multiple fractures as independent fractures, with the exception of those in the fingers, toes, metacarpals, and 2 fractures in the same bone, which were categorized as a single fracture. Fractures of skull, sternum, teeth, nose, and ribs were not included. This ascertainment method with chart reviews allowed us to avoid double counting of fractures (due to multiple visits or numerous sequential radiographs).

Through the use of medical records we registered age, sex, date of fracture, fractured region, fractured side, trauma mechanism, trauma activity, and trauma severity (Landin 1983, Tiderius et al. 1999, Lempesis et al. 2017). Trauma severity was, as in previous reports, classified as slight, moderate, or severe. Slight injuries included falls from heights less than 0.5 meters (m) and most sport injuries. Moderate injuries involved falls from heights between 0.5 and 3 m, bicycle injuries, falls from swings and slides, and falls downstairs. Severe injuries comprised falls from heights above 3 m and traffic injuries with a motor vehicle involved. Trauma mechanism was classified into falls, mechanical force (including caught or squeezed, bites, blows, and hit by moving object), non-classifiable, or unknown. We also classified etiology of the fractures during 2014–2016 according to the NOMESCO Classification of External Causes of Injuries (Jørgensen et al. 2007).

### Validation

To validate the fracture ascertainment method, 1 of the authors (VL) reviewed all digital skeletal radiographs in Malmö on children under the age of 17 during the period from January 1, 2005 to February 28, 2005 and found 103 fractures. During the same 2 months, through the digital hospital in- and outpatient records used in this report, we also found 103 fractures. Both methods identified 100 fractures, while 3 fractures were identified only by 1 of the methods, indicating a misclassification rate of 3% (Lempesis et al. 2017).

### Statistics

SPSS (IBM SPSS Statistics 24; IBM Corp, Armonk, NY, USA) and Microsoft Excel 2016 (Microsoft Corp, Redmond, WA, USA) were used for statistical calculations. Data are presented as numbers, proportions (%), and incidences per 10^5^ person-years. We used direct standardization with the average population of the city of Malmö during the examined period (in 1-year classes) as reference, to estimate age-adjusted rates. Fracture registration was grouped in the following years: 1950, 1955, 1960, 1965, 1970, 1975–1979, 1993–1994, 2005–2006, and 2014–2016. All evaluated years during a 10-year period were included when calculating the incidences during that specific decade. Difference in fracture incidence between 2 decades are assessed by incident rate ratios (IRRs) with 95% confidence intervals (CI). Trend changes are estimated as mean (CI) annual percentage changes (APC) in fracture incidence, determined by use of joinpoint regression analysis (Kim et al. 2000, Joinpoint Regression Program 2019). We used fracture incidence for each evaluated year (in total 17 points) in the analyses. We considered p < 0.05 to represent a statistically significant difference.

### Ethics, funding, and potential conflicts of interest

The Regional Ethical Review Board in Lund approved the study (reference number 2016/1080). The study was organized and performed according to the Declaration of Helsinki.

Financial support for the study was provided by ALF, the Herman Järnhardt Foundation, the Greta and Johan Kock Foundation, and Region Skåne FoU. The sources of funding were not involved in the design or in the conduct of the study, in the interpretation of data, or in the writing of the manuscript. None of the authors have any competing interests.

## Results

### Fracture epidemiology 2014–2016

During the years 2014–2016 we identified 3,244 fractures (61% in boys) during 181,617 person-years. This represents a fracture incidence of 1,786 per 10^5^ person-years (2,135 in boys and 1,423 in girls) (Table 1). In this cohort, 2,743 children had sustained 1 fracture and 236 children (8%) had sustained 2 or more fractures. These 236 children (171 boys) with multiple fractures had sustained 501 fractures; 214 had sustained 2 fractures, 16 had sustained 3 fractures, 5 had sustained 4 fractures, and the remaining 5 fractures were sustained by a single child (falling injury).

**Table 1.** Number of fractures, fracture incidences (per 10^5^ person-years), and proportions (%) of fractures in all children, boys and girls aged 0–15 years in Malmö, Sweden, during the years 2014–2016

	All children			Boys			Girls		
	Number	Incidence	Proportion	Number	Incidence	Proportion	Number	Incidence	Proportion
All fractures	3,244	1,786	100	1,978	2,135	100	1,266	1,423	100
Axial	29	16	0.9	14	15	0.7	15	17	1.2
Face	12	7	0.4	7	8	0.4	5	6	0.4
Spine	8	4	0.2	3	3	0.2	5	6	0.4
Pelvis	9	5	0.3	4	4	0.2	5	6	0.4
Extremity	3,215	1,770	99	1,964	2,120	99	1,251	1,406	99
Upper extremity	2,520	1,388	78	1,562	1,686	79	958	1,077	76
Scapula	1	1	0.0	0	0	0.0	1	1	0.1
Clavicle	258	142	8.0	169	182	8.5	89	100	7.0
Humerus	358	197	11	183	198	9.3	175	197	14
Proximal	82	45	2.5	40	43	2.0	42	47	3.3
Diaphyseal	13	7	0.4	5	5	0.3	8	9	0.6
Distal	263	145	8.1	138	149	7.0	125	140	9.9
Forearm	1,288	709	40	791	854	40	497	558	39
Proximal	94	52	2.9	51	55	2.6	43	48	3.4
Diaphyseal	203	112	6.3	129	139	6.5	74	83	5.8
Distal	991	546	31	611	660	31	380	427	30
Hand	615	339	19	419	452	21	196	220	15
Carpal and metacarpal	175	96	5.4	145	157	7.3	30	34	2.4
Finger	440	242	14	274	296	14	166	187	13
Lower extremity	695	383	21	402	434	20	293	329	23
Femur	42	23	1.3	25	27	1.3	17	19	1.3
Proximal	4	2	0.1	0	0	0.0	4	4	0.3
Diaphyseal	25	14	0.8	19	21	1.0	6	7	0.5
Distal	13	7	0.4	6	6	0.3	7	8	0.6
Patella	10	6	0.3	6	6	0.3	4	4	0.3
Tibia	278	153	8.6	153	165	7.7	125	140	9.9
Proximal	42	23	1.3	30	32	1.5	12	13	0.9
Diaphyseal	176	97	5.4	92	99	4.7	84	94	6.6
Distal	60	33	1.8	31	33	1.6	29	33	2.3
Fibula	44	24	1.4	24	26	1.2	20	22	1.6
Proximal and diaphyseal	2	1	0.1	2	2	0.1	0	0	0.0
Distal	42	23	1.3	22	24	1.1	20	22	1.6
Foot	321	177	9.9	194	209	9.8	127	143	10
Mid- and hindfoot	11	6	0.3	7	8	0.4	4	4	0.3
Metatarsals	171	94	5.3	103	111	5.2	68	76	5.4
Toe	139	77	4.3	84	91	4.2	55	62	4.3

**Figure 1.**
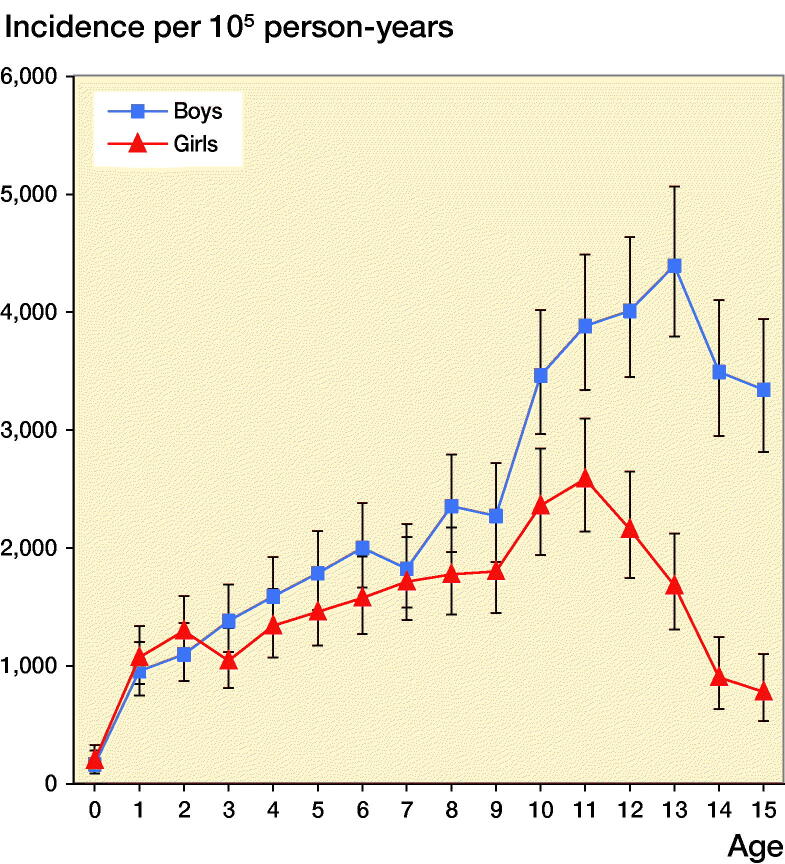
Fracture incidence per 10^5^ person-years in boys and girls aged 0–15 years in Malmц, Sweden, during the years 2014–2016. Data are presented with 95% confidence intervals (CI 95%) per 1-year age class.

The age-adjusted boy-to-girl incident rate ratio was 1.6 (CI 1.5–1.7). Before age 10 years, we found no statistically significant sex differences between boys and girls in 1-year age classes (except at age 8). Boys from age 10, however, had a higher fracture incidence than girls, with the greatest discrepancy found at age 15, with a boy-to-girl fracture IRR of 4.3 (CI 2.9–6.2). The peak fracture incidence in boys was reached at age 13 (4,396 per 10^5^ person-years) and in girls at age 11 (2,585 per 10^5^ person-years) (Figures 1 and 2, see Supplementary data). The peak fracture incidence in 2014–2016 in boys occurred at similar age to 1950/1955 and 1 year earlier than in 2005–2006 and was at a similar magnitude to 2005–2006. The peak fracture incidence in girls in 2014–2016 occurred about 1 year earlier than in 1950/1955 and 2005–2006 and the magnitude was higher than in 1950/1955 and 2005–2006 (Figure 3).

Figure 3.Fracture incidence per 10^5^ person-years in boys (left panel) and girls (right panel) aged 0–15 years in Malmц, Sweden, during the periods 1950/1955 (study start), 2005–2006 (earlier study), and 2014–2016 (present study), representing fracture incidence during different decades.

**Figure:**
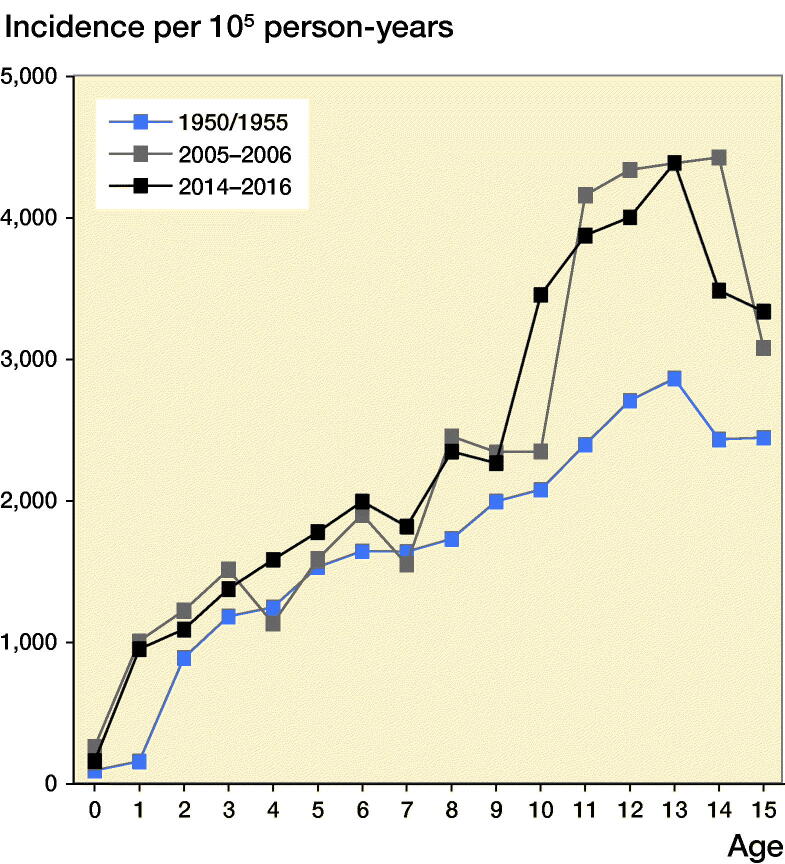

**Figure:**
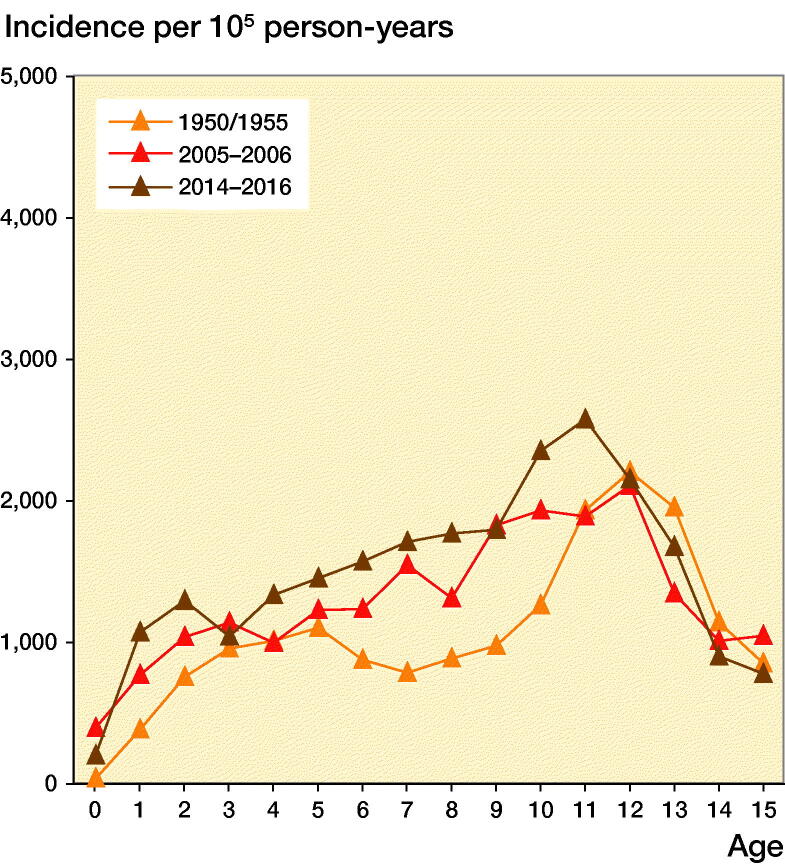

78% of all fractures occurred in the upper extremities, 21% in the lower extremities, and 1% in the axial skeleton. Of all fractures 31% were distal forearm fractures, 14% finger fractures, and 8% distal humerus fractures (Table 1). Fractures were 22% more common in the left than the right arm (IRR = 1.2, CI 1.1–1.3), while we found no apparent left to right difference in the legs (IRR = 0.9, CI 0.8–1.05).

We registered the highest incidences in May (214 per 10^5^ person-years), September (209), and June (183). The lowest incidences were found in December (90 per 10^5^ person-years), January (107), and November (109) (Figure 4, see Supplementary data).

### Fracture epidemiology 1950–2016

Both the unadjusted and age-adjusted fracture incidences were in all children, as well as in boys and girls separately, higher in 2014–2016 than in 1950/1955 and 1960/1965. The unadjusted and age-adjusted incidence in girls in 2014–2016 was also higher than in 2005–2006. In contrast, the unadjusted fracture incidence in 2014–2016 was in all individuals, as well as boys and girls separately, lower than in 1970/1975–1979 (Table 2, Figure 5).

Figure 5.Unadjusted fracture (left panel) and age-adjusted fracture (right panel) incidence per 10^5^ person-years in boys and girls aged 0–15 years in Malmц, Sweden, in 2014–2016 in comparison with 1950/1955, 1960/1965, 1970/1975–1979, 1993–1994, and 2005–2006. These periods are shown with thick lines, with line markers representing the number of years measured in the period. An arrow indicates the 2 compared periods with incident rate ratios (IRR) in fracture incidence with 95% confidence intervals (95% CI) presented above the arrow. **^a^**indicates statistically significant changes.

**Figure:**
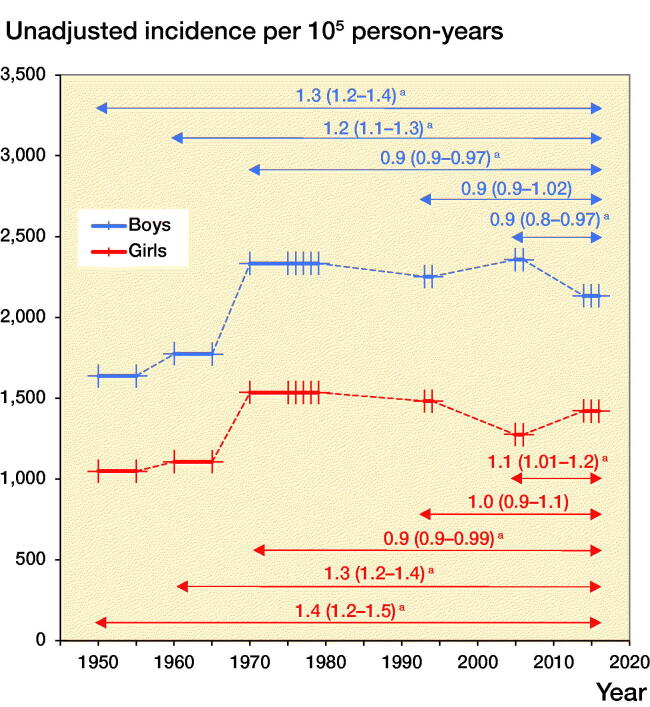

**Figure:**
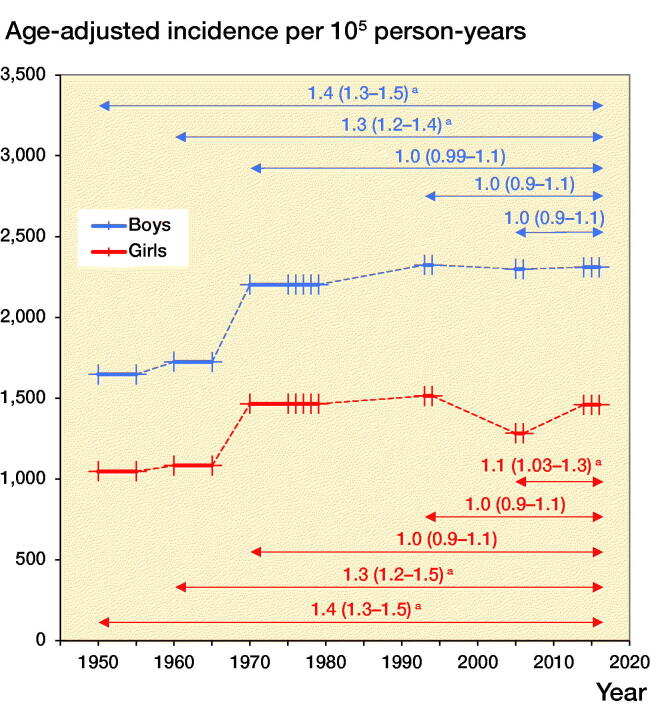

**Table 2.** Unadjusted and age-adjusted fracture incidence differences presented as incident rate ratios with 95% confidence intervals between periods of interest (2014–2016 in comparison with 1950/1955, 1960/1965, 1970/1975–1979, 1993–1994, 2005–2006) in all children aged 0–15 years in Malmö, Sweden

Nominator	2014–2016	2014–2016	2014–2016	2014–2016	2014–2016
Denominator	1950/1955	1960/1965	1970/1975–1979	1993–1994	2005–2006
Unadjusted	1.32 (1.24–1.41) ^a^	1.23 (1.16–1.31) ^a^	0.92 (0.88–0.96) ^a^	0.95 (0.89–1.01)	0.97 (0.92–1.03)
Age-adjusted	1.40 (1.31–1.49) ^a^	1.34 (1.26–1.43) ^a^	1.03 (0.98–1.07)	0.98 (0.92–1.04)	1.05 (0.99–1.11)

**^a^**Statistically significant changes.

**Figure 6.**
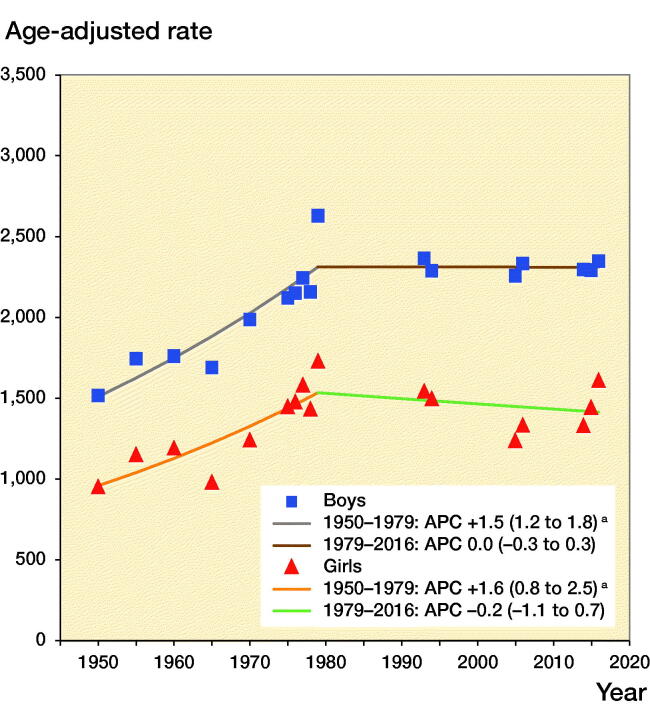
Age-adjusted fracture incidence per 10^5^ person years in boys and girls aged 0–15 in Malmц, Sweden, during the years 1950–2016, estimated with joinpoint regression. **^a^**indicates statistically significant changes.

From 1950 to 1979 age-adjusted fracture incidence increased in boys by +1.5% per year (APC +1.5%, CI 1.2–1.8) and in girls by +1.6% (APC +1.6%, CI 0.8–2.5). From 1979 to 2016 fracture incidence was stable in both boys (APC 0.0%, CI –0.3 to 0.3) and girls (APC –0.2%, CI –1.1 to 0.7) (Figure 6). We must, however, acknowledge that different fractures may have different time trends. The proportion of diaphyseal femur fractures for example halved from 1.6% in 1950–1979 to 0.8% in 2014–2016, whereas the proportion of diaphyseal forearm fractures almost doubled, from 3.4% in 1950–1979 to 6.3% in 2014–2016.

### Fracture etiology 2014–2016

Fracture etiology is presented according to the Landin classification (Landin 1983) (Table 3, see Supplementary data) and the NOMESCO classification (Table 4, see Supplementary data). Using the NOMESCO classification 28% of all fractures occurred during sports activity and 29% during playing activity. The corresponding proportions according to Landin classification were 24% and 23%, respectively. Of all fractures, 16% occurred during ball games (Table 3, see Supplementary data). Using NOMESCO the cause of injury can be further specified. Of the ball game fractures, 78% were sustained during football and 7% during basketball. Among the fractures in the category contact sport (such as wrestling, boxing, and taekwondo) (Table 3, see Supplementary data) 35% occurred during taekwondo. The most common location for fights was in school (with 29% of all fights). Of all fractures that occurred in school, 45% were sustained during sports activity and 18% during playing activity (Table 4, see Supplementary data).

### Fracture etiology 1950–2016

The most common trauma mechanism in children during every time period was a fall on the same plane, and except for 1960/1965 the most common trauma-related activity was sports injuries (Table 3, see Supplementary data).

## Discussion

### Fracture epidemiology 2014–2016

We found a pediatric fracture incidence in 2014–2016 of 1,786 per 10^5^ person-years, slightly lower than the fracture incidence of 2,050 per 10^5^ person-years in Norway in 2010–2011 (Christoffersen et al. 2016). This difference could partly be due to the fact that the studies use different fracture ascertainment methods and that the countries have a differing winter climate and as well as different common leisure-time activities. This view is supported when finding a pediatric fracture incidence of 2,230–2,240 per 10^5^ person-years in northern Sweden in 2005–2006 (Hedström et al. 2010) in comparison with 1,832 per 10^5^ person-years in the south of Sweden during the same period (Lempesis et al. 2017). However, when comparing fracture incidences between different regions it is also important to take into account the proportion of boys and girls, distribution of children between cities and countryside, and proportion of immigration, all factors that may influence the incidence (Moon et al. 2016, Lempesis et al. 2017).

The peak fracture incidence in 2014–2016 occurred at age 13 for boys and age 11 in girls. Most studies infer that peak fracture incidence coincides with puberty (Faulkner et al. 2006, Hedström et al. 2010, Mayranpaa et al. 2010) and current data also infer that peak fracture incidence in 2014–2016 occurs in younger ages compared with previous decades. We speculate that one of the reasons for this could be that puberty seems to start earlier nowadays than historically (Brix et al. 2019). The current data also support previous publications that pediatric fractures occur more often in the upper than lower extremity (Tiderius et al. 1999, Lempesis et al. 2017), more often in the left than in the right arm, more often in the non-dominant than in the dominant arm (Tiderius et al. 1999, Anjum et al. 2017, Lempesis et al. 2017) and with a higher incidence during the warmer than during the colder season (Tiderius et al. 1999, Hedström et al. 2010).

### Time trends

Both boys and girls had higher unadjusted and age-adjusted incidence in 2014–2016 than in 1950/1955 and 1960/1965, indicating that changes in demographics could not entirely explain these higher fracture incidences in the recent period. During these periods, there may also have been different proportions of children living in rural or urban areas, different sports habits, different patterns regarding seeking medical advice, and differing availability of health care (Landin 1983). The efficacy of traffic safety work in Sweden during the decades examined is supported in this study with lower proportions of traffic injuries during recent years. The large influx of pediatric immigrants in Sweden in recent years (Statistics Sweden 2020), the gradual decrease in physical activity during the most recent decades, and the dramatic increase in sedentary screen time activities since 2010 (Swedish Media Council 2017, Raustorp et al. 2019) may be other important contributors.

A problem with previous published studies (Landin 1983, Tiderius et al. 1999, Hedström et al. 2010, Mayranpaa et al. 2010, Lempesis et al. 2017, Koga et al. 2018) is that these have mainly compared 2 incidences between different years (or periods), not taking the natural variation between years (or periods) into account. For example, if in the study by Lempesis et al. (2017) the year 1970 is chosen to represent unadjusted fracture incidence in this decade, the fracture incidence 2005 was 9% higher, while if instead the year 1979 is used the fracture incidence in 2005 was 23% lower. That is, simply by choosing a different year within the same decade, the natural variation in fracture incidence could lead to different conclusion. A regression using multiple observations may thus be more suitable but may still be inadequate if changes in trends during the period of examination are prevalent. Joinpoint regression takes possible changes in trends into account and with this analysis we were able to examine the entire period from 1950 to 2016. We then found that the annual fracture incidence increased from 1950 to 1979 in both boys and girls, similar to the conclusions drawn by Landin (1983), who compared only 2 periods. However, we also found stable incidences thereafter (from 1979 to 2016) in both boys and girls, which contradicts the conclusions by Tiderius et al. (1999), who compared fracture incidence in 1975–1979 with 1993–1994 and Lempesis et al. (2017) who compared fracture incidence in 1976–1979 with 2005–2006. This highlights that time-trend inferences should be interpreted with care when based on comparison between 2 defined periods.

### Strengths and limitations

Study strengths include the long study period with the same fracture classification system, the availability of official annual population data, the validation of the ascertainment method (used from 2005), inclusion of only objective verified fractures, and the possibility to preclude double counting of the same fracture. Another strength is inclusion of the more detailed NOMESCO classification as a complement to the Landin classification. The use of joinpoint regression analyses for estimation of time trends, instead of comparing incidences between 2 periods, is also a study strength.

Weaknesses include the use of 2 different ascertainment methods, 1 from 1950 to 1994 and 1 from 2005 to 2016, and that children living in Malmö but treated and followed up at other hospitals, in previous validation studies found to represent 3%–7% misclassification of fractures (Jonsson 1993), would not be registered by our method. The large proportion of unknown trauma (varying from 25% to 54% in different periods) is another weakness that led us to present only descriptive etiology data. A problem with the etiology data is that these are derived from the medical charts, referrals, and reports of radiographs with sometimes limited or conflicting information. It would have been advantageous to improve the classification system regarding the trauma information in the medical charts. It would also have been advantageous to adjust for changes in ethnicity in the population; however, such data were not available.

## Conclusion

The pediatric fracture incidence in Malmö, Sweden increased from 1950 to 1979, but thereafter was stable until 2016. The higher female incidence in 2014–2016 than in 2005–2006 indicates the necessity to follow fracture incidence continuously during the coming years to reveal any emerging time trends in fracture incidence. This study also highlights the problem when, as in most previous publications, drawing inferences regarding time trends without using multiple observation points.

## Supplementary Material

Supplemental Material

## Supplementary data

Tables 3 and 4 and Figures 2 and 4 are available as supplementary data in the online version of this article, http://dx.doi.org/10.1080/17453674.2020.1783484

The study design was done by EB, VL, BR, and MK. EB collected the data. VL re-reviewed some of the radiographs. LL, CJT, and VL undertook the previous collection of the historical data. EB made the calculations with statistical calculation assistance from JÅN and LJ. EB wrote the first draft of the manuscript. EB, VL, BR, MK, JÅN, and LJ completed the manuscript.

The authors would like to thank Lennart Landin and Carl-Johan Tiderius who conducted the studies 1950–1979 and 1993–1994 respectively.

*Acta* thanks Johannes Mayr and Jaakko Sinikumpu for help with peer review of this study.
