# Supplementary material for: Time trends in pediatric fractures in a Swedish city from 1950 to 2016
Source: Acta Orthop. 2020 Jun 26;91(5):598–604. doi: 10.1080/17453674.2020.1783484 (PMC8023972; doi:10.1080/17453674.2020.1783484)
Supplement: Supplemental Material [file IORT_A_1783484_SM2745.pdf]

Supplementary data

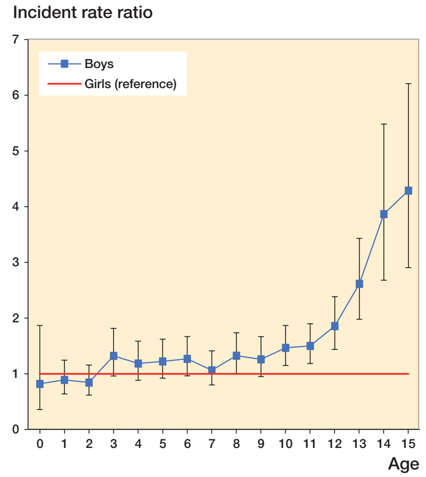

Figure 2. Boy-to-girl incident rate ratio for fracture incidence, in children aged 0–15 years in Malmö, Sweden, during the years 2014–2016. Data are presented with 95% confidence intervals (CI 95%) per 1-year age class.

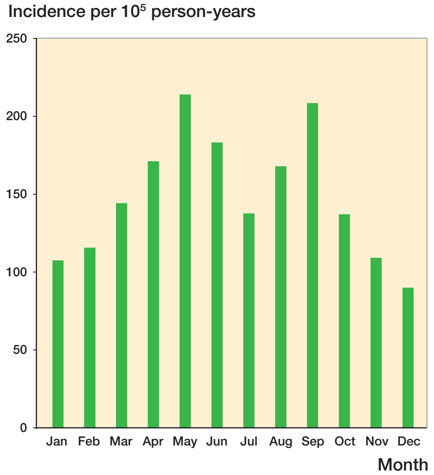

Figure 4. Seasonal variation throughout 2014–2016 in fracture incidence (per 10^5 person-years) in children aged 0–15 years in Malmö, Sweden.

Table 3. Pediatric fracture etiology in Malmö, Sweden, in children aged 0–15 years from 1950/1955 to 2014–2016, regarding trauma activity, trauma mechanism, and trauma severity. Data are presented as proportions (%) of all fractures. No studies were carried out during 1980–1989

	1950/ 1955	1960/ 1965	1970/ 1975– 1979	1993– 1994	2005– 2006	2014– 2016
**Trauma activity**						
Home	5	6	5	7	2	5
Day nursery	0	0	1	2	1	5
School	4	4	4	3	7	10
Work	0	0	0	0	0	0
Traffic injuries	11	13	11	12	9	6
Bicycle	8	5	7	8	7	5
Pedestrian hit by vehicle	2	5	2	1	1	0
Moped, motorcycle	0	1	1	1	1	0
Car passenger	0	1	1	1	0	0
Other	0	1	0	1	0	0
Playing injuries	12	15	15	17	19	23
Playground	3	3	4	7	9	9
In-line skates, skateboard	0	0	1	2	4	5
Sledge, other “snow”	1	0	2	1	2	1
Other	8	11	9	7	5	9
Sports injuries	12	13	19	22	28	24
Ball game	4	5	9	10	17	16
Ice-hockey, skating	6	5	3	3	2	1
Gymnastics and athletics	1	1	1	3	2	1
Horse injuries	1	1	3	3	2	1
Wrestling, boxing, etc.						
“Contact sport”	0	1	1	2	1	1
Skiing	0	1	2	1	3	2
Other	0	0	0	1	1	1
Fights	1	2	3	3	5	1
Other	0	0	2	0	0	1
Unknown	54	48	40	34	29	25
**Trauma mechanism**						
Falls	70	74	79	67	68	66
On the same plane	50	50	56	41	42	35
Between planes	20	25	23	26	26	31
Mechanical force	14	16	15	23	24	24
Non-classifiable	0	1	1	5	8	4
Unknown	16	9	5	4	0	6
**Trauma severity**						
Slight	55	55	65	64	65	64
Moderate	21	22	21	30	25	24
Severe	4	8	6	4	4	2
Non-classifiable	5	6	5	0	4	6
Unknown	15	9	4	2	2	4

 Acta Orthopaedica 2020; 91 (DOI: 10.1080/17453674.2020.1783484)

Table 4A. Etiology of fractures 2014–2016 in children aged 0–15 years in Malmö, Sweden based on the NOMESCO classification presented in defined places with activity that resulted in a fracture. Data are proportions (%)

	Place							
Activity	Home	Day care	School	Sports area	Playing area	Other	Unknown	Total
Sports	1	0	45	97	0	0	1	28
Playing	28	51	18	1	99	3	34	29
Other	10	0	2	0	0	93	3	8
Unknown	61	49	34	1	1	4	63	34
Total	100	100	100	100	100	100	100	100

Table 4B. See Table 4A. Data presented in defined activities where the fracture occurred. Data are proportions (%)

	Activity				
Place	Sports	Playing	Other	Unknown	Total
Home	0	5	6	9	5
Day care	0	9	0	8	5
School	16	6	2	10	10
Sports area	83	1	0	1	24
Play area	0	34	0	0	10
Other	0	1	78	1	7
Unknown	1	44	14	71	39
Total	100	100	100	100	100
